# Pulmonary thromboembolism: retrospective study of necropsies performed over 24 years in a university hospital in Brazil

**DOI:** 10.1590/S1516-31802002000400003

**Published:** 2002-07-07

**Authors:** Valdir Golin, Sandra Regina Schwarzwälder Sprovieri, Rubens Bedrikow, Mauro José Costa Salles

**Keywords:** Pulmonary thromboembolism, Necropsies, Lethal, Tromboembolismo pulmonar, Necropsias, Letal

## Abstract

**CONTEXT:**

Pulmonary thromboembolism is the third most frequent cause of morbidity and mortality among acute cardiovascular diseases. The incidence of pulmonary embolism in necropsies has remained unchanged over the last few decades. Cardiac diseases, neoplasia, trauma, recent surgery and systemic diseases are important predisposing clinical conditions. The relationship between male and female sexes is estimated at 1.24. Various studies have shown an increase in morbidity in spring and autumn. There is great difficulty in precise anatomopathological diagnosis in relation to the localization of the emboli in the pulmonary vessels, although they are preferentially located in the right lung and lower lobes.

**OBJECTIVE:**

To study the incidence of lethal and non-lethal pulmonary thromboembolism in relation to epidemiological and anatomical variables.

**DESIGN:**

Retrospective study performed via reports on the necropsy findings.

**SETTING:**

University hospital providing tertiary-level attendance.

**SAMPLE:**

16,466 consecutive necropsies performed from January 1972 to December 1995.

**MAIN MEASUREMENTS:**

Frequency of lethal and non- lethal pulmonary thromboembolism, predisposing diseases, occurrence in relation to the seasons of the year, and location where the embolus is lodged.

**RESULTS:**

Pulmonary thromboembolism was found in 4.7% of all the necropsies performed. There was a predominance of lethal cases (68.2%). There was no difference in relation to sex or seasons of the year for the occurrence of this disease. Cardiovascular diseases were more frequently associated with thromboembolic phenomena. With regard to the location where the embolus was lodged, various lung segments showed greater incidence of being bilaterally compromised.

**CONCLUSION:**

Over the period of this study, it was observed that there was a reduction in the incidence of pulmonary thromboembolism, which was probably due to the increase in prophylactic measures over the last few decades. Nonetheless, lethal thromboembolism predominated in frequency, probably because of the abrupt onset of a condition of attack across a large area of the lung, lack of clinical suspicions and consequently a lack of early diagnosis, and delay in instituting fibrinolytic therapy in the cases with hemodynamic repercussions or a large number of lung segments affected.

## RESUMO

**CONTEXTO:**

O tromboembolismo pulmonar é a terceira doença cardiovascular aguda mais freqüente como causa de morbidade e mor-talidade. A incidência de embolia pulmonar em necropsias não se modificou nas últimas décadas. Doenças cardíacas, neoplasias, traumas, cirurgias recentes e doenças sistêmicas são condições clínicas predisponentes importantes. A relação entre os sexos masculino e feminino é estimada em 1,24. Vários estu- dos mostram aumento da morbidade no inverno. Há grande dificuldade para o diagnóstico anátomo-patológico preciso quanto a localização dos êmbolos nos vasos pulmonares, havendo preferência por localizarem-se no pulmão direito e lobos inferiores.

**OBJETIVO:**

Estudar a incidência de tromboem- bolismo pulmonar letal e não letal, de acordo com variáveis epidemiológicas e anatômicas.

**TIPO DE ESTUDO:**

Estudo retrospectivo realizado através relatórios dos laudos de necropsias.

**LOCAL:**

Hospital Universitário de atendimento terciário.

**AMOSTRA:**

16.466 necropsias consecutivas realizadas de janeiro de 1972 a dezembro de 1995.

**VARIÁVEIS ESTUDADAS:**

Freqüência de tromboembolismo pulmonar letal e não letal, doenças predisponentes, ocorrência de acordo com as estações do ano e o local de impactação do êmbolo.

**RESULTADOS:**

O tromboembolismo pulmonar foi encontrado em 4,7% de todas as necropsias realizadas. Houve predomínio dos casos letais (68,2%). Não houve diferença em relação ao sexo ou estações do ano para a ocor-rência desta doença. As doenças cardiovasculares estiveram mais freqüentemente asso-ciadas aos fenômenos tromboembólicos. Quanto ao local de impactação do êmbolo, houve maior incidência no comprometimen-to bilateral de vários segmentos dos pulmões.

**CONCLUSÃO:**

Durante o período deste estudo observou-se que houve redução na incidên-cia de tromboembolismo pulmonar, prova-velmente pelo aumento de medidas profiláticas nas últimas décadas. Contudo, a fre- qüência de tromboembolismo letal predomi- nou, provavelmente em função da instalação abrupta do quadro com acometimento de grande área pulmonar, falta de suspeita clínica e portanto do diagnóstico precoce e, demora da instituição de terapêutica fibri-nolítica nos casos com repercussão hemo- dinâmica ou com grande número de segmentos pulmonares afetados.

## INTRODUCTION

Pulmonary thromboembolism is a disease with a high mortality rate, only ranking behind cardiac diseases and cerebral ischemia. In addition to this, the disease is clinically suspected and diagnosed in less than 30% of patients who progress to death.^1-5^

Guerra et al., studying necropsies performed at the Anatomopathological Department of the Faculty of Medical Sciences of Santa Casa, São Paulo, observed that over the period from 1952 to 1961 the incidence of pulmonary thromboembolism was 4.7%.^6^ Amary et al. found a pulmonary thromboembolism incidence of 6% when studying the reports on necropsies performed in the same department by the same medical team between 1962 and 1971.^7^

After the end of the 1970s there was an intensification of prophylactic measures against thromboembolic phenomena. Nevertheless, despite all the advances, mortality caused by this disease did not diminish.^8-10^

Thus, the objective of this study was to evaluate the incidence of pulmonary thromboembolism in necropsies performed during the period from 1972 to 1995. It also had the purpose of verifying the locations where the emboli were lodged, associated diseases, whether there was any influence from the season of the year, the incidence of pulmonary thromboembolism as the immediate cause of death, or whether pulmonary thromboembolism was present only as a secondary disease that was not directly linked to the death.

## METHODS

We made a retrospective and sequential study of the macroscopic and microscopic reports on necropsies performed from January 1972 to December 1995 at the Anatomopathological Department of the Faculty of Medical Sciences of Santa Casa de Misericórdia, São Paulo. The study followed the same techniques and routine employed in the previous studies.

From the total number of reports produced over this period, our review selected the ones in which the main cause of death was diagnosed as pulmonary thromboembolism, denominated Lethal pulmonary thromboembolism, and also the reports in which pulmonary thromboembolism was associated with other diseases that were the direct cause of death, denominated Non-Lethal pulmonary thromboembolism.

Thus, the following items were studied:

1. Anatomopathological data: With regard to the localization of the emboli in the lungs, we considered them as Single when they only affected one lung segment or lobe and Multiple when they attacked more than one segment or lobe;
2. Personal data: sex, age;
3. Season of the year when death occurred;
4. Diseases associated with or considered as causing a risk of thromboembolism.

### Statistical analysis

The data obtained were stored in the databank program within EpiInfo. The statistical analysis was performed via the EpiInfo Version 6 program (CDC, USA, 1997; WHO, Geneva, Switzerland).

In the comparative study of the non-parametric variables, the Chi-Squared test was utilized. When the proportions indicated a trend, the Chi-Squared test for trends was utilized. The level of significance adopted was 5% (p < 0.05).

## RESULTS

Of the 40,998 deaths that occurred at the Central Hospital of Santa Casa, São Paulo, during the 24 years of the study period (1972-1995), necropsies were performed in 16,466 cases (40%). Among these 16,466 necropsy reports that were reviewed, pulmonary thromboembolism was found in 782 cases (4.7%). Among these cases of pulmonary thromboembolism, it was observed that there were a greater number of cases of Lethal pulmonary thromboembolism pulmonary thromboembolism than of Non-Lethal pulmonary thromboembolism (p = 0.0001). That is, 533 thromboembolic phenomena (68%) were lethal and 249 (32%) were non-lethal, with a 95% confidence interval (CI) (Table 1).

**Table 1:** Incidence of pulmonary thromboembolism in necropsies

	Period 1972-1995	95% CI
Number of deaths	40,998	
Necropsies	16,466	
Necropsies with PTE	782	
Necropsies with lethal PTE	533*	
Necropsies with non-lethal PTE	249	
Incidence of PTE in necropsies (%)	4.7%	4.4-5.1
Incidence of lethal PTE in necropsies (%)	3.2%*	3.0-3.5
Incidence of non-lethal PTE in necropsies (%)	1.5%	1.3-1.7

* *p = 0.001*

*c^2^ trend = 14.97; 1 gl*

*PTE = pulmonary thromboembolism CI = confidence interval.*

**Figure 1:**
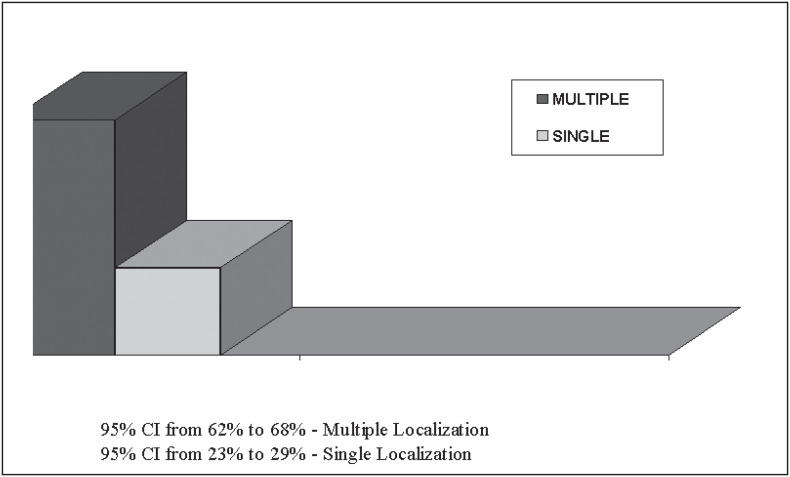
Localization of where emboli were lodged.

Figure 1 shows that the emboli were found preferentially in multiple lung segments or lobes (95% CI 95% from 62% to 68%).

Cardiovascular diseases were the ones most frequently associated with pulmonary thromboembolism, in 65% of the cases (95% CI from 62% to 68%). In second place were the infectious diseases, in 27% of the cases (95% CI from 24% to 29%), and then came, in sequence, postoperative cases, in 24% of the cases (95% CI from 21% to 27%), neoplasia, in 18% of the cases (95% CI from 15% to 21%), and finally the chronic obstructive pulmonary diseases, in 17% of the cases (95% CI from 15% to 20%) (Table 2). The average age of the patients was 55 ± 20.2 years, with 56.8% of the cases falling within the age range of 50-79 years.

**Table 2:** Incidence of the most frequent diseases in necropsies with thromboembolism

Associated diseases	%	95% CI
Cardiovascular	65%	62-68
Infectious	27%	24-29
Postoperative	24%	21-27
Neoplasia	18%	15-21
Chronic pulmonary disease	17%	15-20

*CI = confidence interval.*

No difference was observed in the frequency of pulmonary thromboembolism between the male and female sexes (p = 0.91). There was no difference in the frequency of thromboembolism between the seasons of the year (p = 0.06).

## DISCUSSION

The data obtained showed that the incidence of pulmonary embolism in the necropsies performed was 4.7%, with a predominance of cases of Lethal pulmonary thromboembolism in relation to Non-Lethal pulmonary thromboembolism (Table 1). With regard to the emboli, they were more frequently found in multiple regions of the lungs (Figure 1). The disease that was most associated with pulmonary thromboembolism was cardiovascular disease, followed by infectious disease, postoperative infections, neoplasia and chronic obstructive pulmonary diseases (Table 2). We did not observe a difference in prevalence in relation to sex, nor did we for the different climatic seasons of the year.

Guerra et al., in this same hospital, observed that the incidence of pulmonary thromboembolism was 4.7% from 1952 to 1963, of which 2.7% was lethal pulmonary thromboembolism.^6^ On the other hand, Amary et al., also at Santa Casa in São Paulo, verified that from 1962 to 1972 the incidence of pulmonary thromboembolism was 6.0%, of which 4.4 % was lethal pulmonary thromboembolism.^7^ In 1978, Maffei et al. reviewed 998 necropsied cases at the University Hospital of Botucatu and found a pulmonary thromboembolism rate of 17%, of which 3.7% were fatal.^8^ Bergqvist et al. found a pulmonary thromboembolism rate of 26% among patients who underwent surgery.^11^

Our results for the incidence of pulmonary thromboembolism are very close to those obtained by Guerra in the 1950s, although they are lower than those found by Amary in the 1960s and by Maffei in Botucatu. However, the period studied by Amary coincided with the installation of the Emergency Service in the Hospital, which caused a significant increase in the population of patients attended with serious conditions. Maffei justified the data obtained in his study because of the regional character of his hospital, and probably utilized necropsy techniques that differed from those utilized by the other authors, giving more emphasis to searching for thromboembolic phenomena. In addition, none of these studies showed up differences in the prevalence between the sexes.

Amary and Guerra's studies did not observe any difference in the incidence of pulmonary thromboembolism in different seasons of the year. On the other hand, studies performed in Europe and Asia have shown that the disease is more frequent in the spring and autumn.^10,18,19^

With regard to the diseases associated with pulmonary thromboembolism, we obtained results similar to the majority of studies, or in other words, cardiovascular diseases were in first place as the ones most frequently associated with pulmonary thromboembolism, followed by infectious diseases and finally by neoplasia.^8,13-18^

The differences between the results relating to the seasons of the year point towards evidence that in the regions where the climatic seasons are well defined, such differences in pulmonary thromboembolism incidence can occur. But in a tropical country like Brazil, no such differences have been encountered in any of the studies made here up to the present time.

Despite the evolution in the quality and specificity of complementary examinations over these decades, and also the evolution in therapy, this study clearly shows that the prevalence of lethal pulmonary thromboembolism has not diminished in this hospital since the first study by Guerra et al. One of the reasons for this lack of reduction is perhaps related to tardiness in suspecting the disease or lack of suspicion within this nosological entity, which may have ended up delaying the start of specific therapy. On the other hand, we believe that fibrinolytic therapy has been utilized little in the more serious cases with hemodynamic repercussions, which could in some way have contributed in many cases towards an unfavorable evolution. This fact has led us to alter the focus and guidance relating to the use of this medication in our Emergency Service, and is the subject of a new prospective study in progress.

In conclusion, we can say that the incidence of pulmonary thromboembolism has not diminished with the passage of the years, under the influence of the use of effective prophylactic measures. In our environment, there is no difference in pulmonary thromboembolism incidence in relation to sex or seasons of the year. This study corroborates other data from Brazilian authors regarding localization and the diseases associated with pulmonary thromboembolism. Other studies originating from other regions of Brazil could lead us to an exact idea of what is taking place in terms of the whole of Brazil, considering that the characteristics of the population and geographic conditions are so diverse.
